# Multiscale and Multimodal Optical Imaging of the Ultrastructure of Human Liver Biopsies

**DOI:** 10.3389/fphys.2021.637136

**Published:** 2021-02-17

**Authors:** Cihang Kong, Stefanie Bobe, Christian Pilger, Mario Lachetta, Cristina Ionica Øie, Nils Kirschnick, Viola Mönkemöller, Wolfgang Hübner, Christine Förster, Mark Schüttpelz, Friedemann Kiefer, Thomas Huser, Jan Schulte am Esch

**Affiliations:** ^1^Department of Physics, Bielefeld University, Bielefeld, Germany; ^2^European Institute for Molecular Imaging, University of Münster, Münster, Germany; ^3^Vascular Biology Research Group, Department of Medical Biology, University of Tromsø – The Arctic University of Norway, Tromsø, Norway; ^4^Forschungsverbund BioMedizin Bielefeld (FBMB), Bielefeld, Germany; ^5^Department of General and Visceral Surgery, Evangelisches Klinikum Bethel gGmbH, University Hospital OWL of the University of Bielefeld, Bielefeld, Germany

**Keywords:** liver biology, liver morphology, liver sinusoids, light-sheet fluorescence microscopy, coherent Raman scattering microscopy, super-resolution optical microscopy, liver sinusoidal endothelial cells.

## Abstract

The liver as the largest organ in the human body is composed of a complex macroscopic and microscopic architecture that supports its indispensable function to maintain physiological homeostasis. Optical imaging of the human liver is particularly challenging because of the need to cover length scales across 7 orders of magnitude (from the centimeter scale to the nanometer scale) in order to fully assess the ultrastructure of the entire organ down to the subcellular scale and probe its physiological function. This task becomes even more challenging the deeper within the organ one hopes to image, because of the strong absorption and scattering of visible light by the liver. Here, we demonstrate how optical imaging methods utilizing highly specific fluorescent labels, as well as label-free optical methods can seamlessly cover this entire size range in excised, fixed human liver tissue and we exemplify this by reconstructing the biliary tree in three-dimensional space. Imaging of tissue beyond approximately 0.5 mm length requires optical clearing of the human liver. We present the successful use of optical projection tomography and light-sheet fluorescence microscopy to derive information about the liver architecture on the millimeter scale. The intermediate size range is covered using label-free structural and chemically sensitive methods, such as second harmonic generation and coherent anti-Stokes Raman scattering microscopy. Laser-scanning confocal microscopy extends the resolution to the nanoscale, allowing us to ultimately image individual liver sinusoidal endothelial cells and their fenestrations by super-resolution structured illumination microscopy. This allowed us to visualize the human hepatobiliary system in 3D down to the cellular level, which indicates that reticular biliary networks communicate with portal bile ducts via single or a few ductuli. Non-linear optical microscopy enabled us to identify fibrotic regions extending from the portal field to the parenchyma, along with microvesicular steatosis in liver biopsies from an older patient. Lastly, super-resolution microscopy allowed us to visualize and determine the size distribution of fenestrations in human liver sinusoidal endothelial cells for the first time under aqueous conditions. Thus, this proof-of-concept study allows us to demonstrate, how, in combination, these techniques open up a new chapter in liver biopsy analysis.

## Introduction

The human liver is the largest internal organ of the human body and indispensable for the maintenance of physiological homeostasis. Essential functions of the liver include uptake and metabolism of nutrients, synthesis of glycogen, lipids, amino acids and hormones, and the production and secretion of serum proteins including various lipoproteins, albumin and the constituents of the coagulation system. Fast access to dietary components and xenobiotics entering the circulation predestines the liver to a prime role in the uptake and storage of vitamins and metals, carbohydrate metabolism but also detoxification in particular of hydrophobic substances. The liver is the central metabolic hub of any organism and at the same time responsible for the production of bile and digestive factors and the discharge of metabolic end products and solubilized hydrophobic molecules.

**FIGURE 1:**
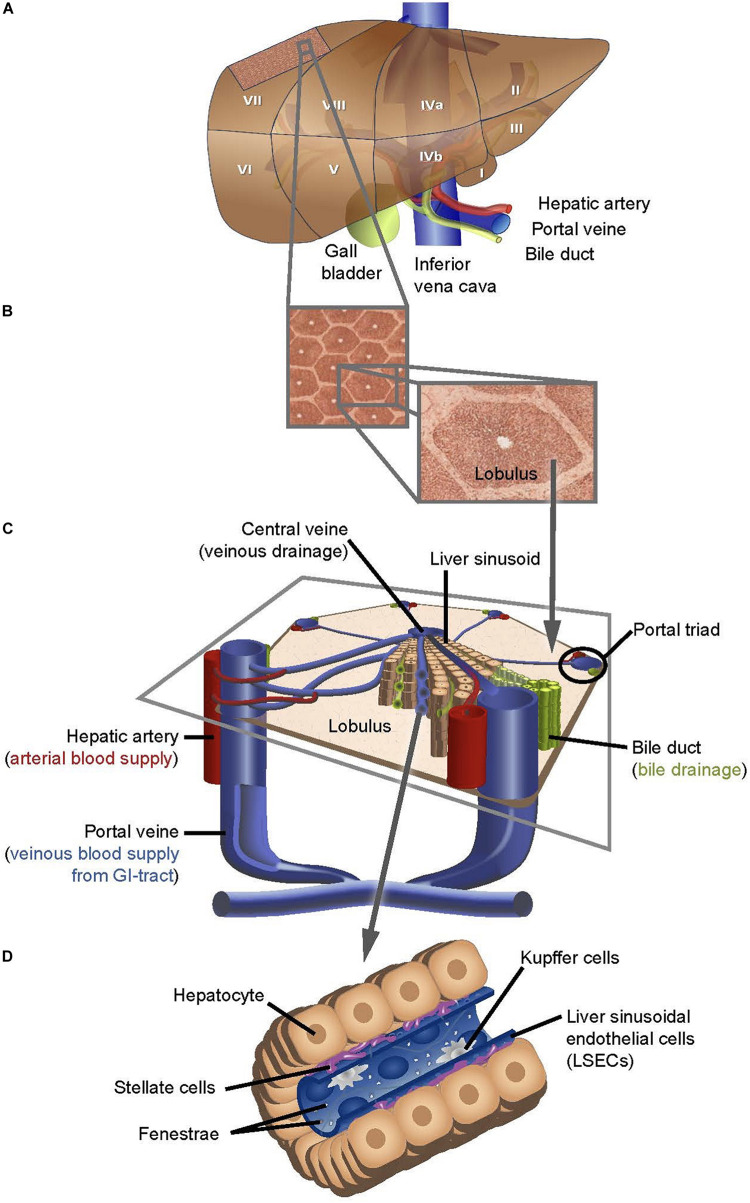
Schematic depiction of the structure of the human liver at different scales of resolution. **(A)** Traditional segmentation used in anatomy and surgery, subdivides the human liver into eight segments. **(B)** Each segment is composed of numerous liver lobules that are packed in a honeycomb pattern (**B**. left panel) and individual lobules are separated by bands rich in extracellular matrix (**B**. right panel). **(C)** Every tripartite junction between liver lobules forms a portal field, composedof a venous vessel originating from a branch of the portal vein, an arterial vessel originating from the hepatic artery and one or more bile ducts. The basic functional unit of the liver is comprised of a central sinusoid flanked by trabeculae of hepatocytes that enclose with their apical membrane a primary bile canaliculus, finally draining into the bile duct in the portal triad. All sinusoids of a lobule drain into a single central venous vessel. **(D)** The distance between the fenestrated endothelial cells that form the sinusoids and the hepatocyte canaliculi is referred to as Space of Disse and contains stellate cells, while Kupffer cells patrol within the endothelial lumen.

The central physiological function and metabolic activity of the liver are reflected by its unique circulatory integration. About 75% of the blood supply to the liver are delivered by the portal vein providing rapid access to newly absorbed dietary constituents. The remaining perfusion is comprised of freshly oxygenated blood provided by the hepatic artery. Inflowing blood is split among the eight segments of the two liver lobes, with each segment possessing its independent vascular supply and extrinsic bile duct (Figure 1A).

Blood is rapidly distributed into smaller caliber vessels that end in hepatic capillaries of 5 to 10 μm diameter, termed sinusoidal vessels, which within a liver lobule drain into the same central vein (Figures 1B,C). Strands of hepatocytes, termed trabeculae, which at their apical junction form bile canaliculi, embed the sinusoidal vessels and together, hepatocytes and endothelial cells constitute the functional units of the liver. Sinusoidal liver endothelial cells (LSECs) are a highly specialized type of endothelium with unique morphology and functions (Sørensen et al., 2015). LSECs contain many small transmembrane pores, or fenestrations, with average diameters of 100 – 150 nm, within a range of 50 – 300 nm (Braet and Wisse, 2002; Øie et al., 2018), which provide open channels between the sinusoidal blood and the subendothelial space, the “Space of Disse,” facilitating the transfer of substrate between the blood and the parenchymal hepatocytes (Figure 1D). A striking functional characteristic of the LSEC is its high endocytic capacity via membrane associated receptors. LSECs do not have a basement membrane. Instead, they face the matrix “Space of Disse,” the abluminal border of which is formed by the basal surface of the surrounding hepatocytes (Figure 1D) (Treyer and Müsch, 2013). LSECs continuously filter plasma via their fenestrae into the Disse space, where it is probed and its components are metabolized by hepatocytes.

The Disse space also harbors hepatic stellate cells, which store, among other lipophilic factors, vitamin A, while the liver-resident macrophages, Kupffer cells, patrol the luminal side of the sinusoidal vessels (Figure 1D). The regular arrangement of the maximum possible number of liver lobules results in a classic honeycomb structure where each lobule is surrounded by six neighboring lobules, characterized by their centrally located veins and a portal field at each tripartite junction of the hexagonal edges (Figures 1B,C). In addition to the portal arteries and veins, portal fields also contain the hepatic lymphatic vessels that were reported to originate as cul-de-sacs.

The apical surface of the hepatocytes form the biliary canaliculi, which at the border to the portal fields merge into the bile duct as part of the portal triad. Liver pathologies directly impact on cholangiocyte structure and function, and may result in cholestasis causing inflammation and liver dysfunction. Conversely, cholangiocyte dysfunction may actively initiate or foster inflammation causing or contributing to liver injury. In particular, the 3-dimensional structure of the transitory zone between bile canaliculi and the more robust intrinsic bile ducts, also called Canal of Hering, has so far not been visualized in the human liver by volume imaging. This transitory zone between the liver parenchyma and the intrinsic biliary tree is partly lined by hepatocytes, partly by small cholangiocytes, and demonstrates the upregulation of EpCAM + hepatic stem/progenitor cells in scenarios of large scale hepatocellular injury with the subsequent ductular reaction (Dollé et al., 2015). Given the important implication of this area in case of dysfunction for the development of liver disease but also for liver regeneration following high-grade liver damage, a detailed structural understanding is highly warranted. Here, we demonstrate how the high level of specificity as well as the deep penetration depth of fluorescent and label-free optical microscopies allow us to image these liver structures across 7 orders of magnitude - from the centimeter scale down to the nanoscale.

## Materials and Methods

### Harvesting of Human Liver Biopsy Samples

Informed consent according to local and international guidelines was signed by all patients. All further experimental procedures were ethically approved (Ethics committee Münster, Germany, 2017-522-f-S). Liver samples of a size of roughly 1 cm^3^ were obtained from a human patient suffering from a hepatically metastasized rectal adeno carcinoma following robotic assisted deep anterior rectal resection 18 months prior to the actual hepatic resection of metachron liver metastases in hepatic segments III and IVa. The liver resection was performed subsequent to an induction of chemotherapy according to the FOLFOXIRI-protocol (11 cycles) with a systemic therapy-free interval of six weeks. Following tissue sampling for routine histopathological purposes, study samples were harvested from a non-neoplasm-involved area of the resected tissue sample (segment III) by an experienced pathologist. Classical hepatic histopathology of the here utilized non-tumor affected liver-tissue revealed no significant pathology. Samples were cut from resected liver tissue and immediately placed in 4% formaldehyde for 1 hour at room temperature for fixation. They were subsequently transferred to a phosphate buffered saline solution containing 0.5% paraformaldehyde for longer term storage followed by specialized sample treatment as required for each specific imaging technique.

### Whole Mount Staining of Human Liver

Human liver tissue was fixed in 4% formaldehyde in phosphate buffered saline (PBS) for 2 h, permeabilized (5% Triton X-100/PBS) and subsequently blocked in Permblock solution (3% BSA, 0.5% Tween-20 in PBS). For whole mount immunostaining anti-cytokeratin 19 (proteintech, number 14965-1-AP), anti-αSMA-Cy3 (Sigma, clone 1A4) and Alexa647-coupled secondary antibody (Molecular Probes) were used in Permblock solution. Antibody incubation was performed for at least 3 weeks at 37°C and samples were washed with PBS-T (0.1% Tween-20/PBS) after each step. The whole mount stained samples were embedded in cylindrical 1% low melting agarose to avoid light scattering at agarose edges during later imaging. Following dehydration and delipidation in increasingly concentrated methanol (70%, 95%, > 99%, > 99%, each step at least 2 h), optical clearing was performed by gradually replacing methanol with a 1:2 benzyl alcohol-benzyl benzoate solution (BABB, Murray’s clear) for refractive index matching. Samples were equilibrated in BABB at least one day and subsequently imaged by light sheet fluorescence microscopy (LSFM) and optical projection tomography (OPT).

### Preparation of Liver Sections for Non-linear Optical Imaging

In order to accommodate the forward-scattering geometry of non-linear optical microscopy methods, such as coherent Raman scattering and second harmonic generation microscopy, where the excitation light is focused into the sample on an inverted microscope and the portion of the light converted to another wavelength within the sample is collected in the forward direction, 50 μm thick sections of liver samples were prepared. Freshly excised liver cubes of approx. 1 cm^3^ volume were stored on ice and were then embedded in TissueTek O.C.T. compound prior to cryo-microtome sectioning. After freezing the tissue blocks were cut to 50 μm thick slices, which were then placed on a glass coverslip (#1, Roth, Karlsruhe). After fixation in 4% formaldehyde for 30 min at RT samples were rinsed with 0.5% formaldehyde. A second cover slip was then placed on top and forms a sandwich around the liver sample for the measurements.

### Isolation and Staining of Human Liver Sinusoidal Endothelial Cells (LSEC)

Liver tissues were obtained from patients undergoing hepatic resections for liver metastasis from colorectal carcinoma. Ethical approval for the study was granted by the Norwegian Research Ethics Committee, and the study protocol conformed to the ethical guidelines of the 1975 Declaration of Helsinki. Written informed consent was obtained from each patient. Human LSECs were isolated based on a method developed at the Vascular Biology Research Group, UiT-The Arctic University of Norway, Tromsø, using Percoll gradient and magnetic-activated bead cell sorting (Øie et al. - manuscript in preparation). The cells were seeded on fibronectin coated 13 mm #1.5 glass bottom dishes (MatTek, Ashland, MA, United States) at a density of 3 × 10^4^ cells/cm^2^. Following attachment and spreading of the cytoplasm, the cells were fixed in 4% formaldehyde, stained and imaged. To visualize fenestrations, the plasma membrane was stained for 10 min with CellMask Orange (1:1000 in PBS).

### Optical Projection Tomography

An optical projection tomography system was built following the description by Nguyen et al. (2017). In essence, this system resembles a widefield-fluorescence microscope using long focal length achromatic lenses for excitation and detection and utilizes a pinhole to extend the depth of field across the sample. Samples as large as 27 mm × 27 mm × 27 mm can be imaged with a spatial resolution down to 30 μm, enabling the examination of entire rodent organs. Samples were rotated within the focus of the system by 360° and images are taken with a step size of 1° or less. To penetrate through the entire sample, optical clearing of the samples is required, though. The only major modification to Nguyen et al. was the use of a different camera. Here, a CMOS camera with 20 MPixels and a pixel size of 2.4 μm was used (Tucsen FL-20BW). For excitation, LED light sources and corresponding filter sets were used as follows: For Alexa488, a filter set containing a 480/30 nm bandpass filter as excitation filter, a 505DC dichroic mirror, and a combination of a 535/50 nm and 520/40 nm bandpass filters as emission filters, for Alexa647, a filter set containing a 625/20 nm bandpass filter as excitation filter, a 650DC dichroic mirror, and a 700/75 nm bandpass filter as emission filter, for Cy3, a filter set containing a 550/60 nm bandpass filter as excitation filter, a 588DC dichroic mirror, and a combination of bandpass and longpass filters (545/30 nm, 593/40 nm bandpass filter in conjunction with a 568 nm longpass filter as detection filter. The system provides a magnification factor of 0.5 with an aperture set to 12 mm diameter providing a depth of focus of approx. 2 mm. For the OPT images shown here, 600 images at a rotation step size of 0.6° were acquired for each color channel. The resulting raw images were reconstructed using a filtered-back projection (FBP) algorithm utilizing a Shepp-Logan filter (Vallejo Ramirez et al., 2019) (reconstruction software:^1^).

### Light Sheet Fluorescence Microscopy (LSFM)

After whole mount immunostaining and optical clearing, liver tissue was imaged using a LaVision UltraMicroscope II (LaVision BioTec) with a step size of 2 μm and either at 1.6-fold or 5-fold magnification. 3D reconstructions of the acquired stacks were visualized and analyzed using the volume rendering software package Voreen (*voreen.uni-muenster.de*) (Meyer-Spradow et al., 2009; Hägerling et al., 2017).

### Non-linear Optical Microscopy (SRS, CARS, SHG)

A custom-built coherent Raman scattering microscope was used for label-free imaging of liver sections. The laser source consisted of a 1032 nm fiber laser (Emerald Engine, APE, Berlin) operating at 80MHz repetition rate with 2 ps pulse length. The frequency-doubled 516 nm beam pumped an optical parametric oscillator (OPO) (Levante Emerald, APE, Germany), producing a beam with tunable wavelength, which was utilized as pump beam in the coherent anti-Stokes Raman scattering (CARS) and stimulated Raman scattering (SRS) experiments. The 1032 nm laser served as non-tunable Stokes wavelength. The pulsed pump and Stokes laser beams were overlapped in time and space by a dichroic mirror and an optical delay stage. The combined beams are then sent into a custom-built laser scanning microscope. Galvanometric scanning mirrors (Cambridge Technology, Galvanometer Optical Scanner, Model 6215H, United States) were utilized to raster scan the laser focus across the sample. A scanning telescope filled the back focal plane of a 60x water immersion objective lens (Olympus UPlanSApo, NA1.2, Olympus, Germany) focusing the beams into the sample. The signal generated in the sample was collected in the forward direction using an oil immersion condenser lens (U-AAC, NA1.4, Olympus, Germany). The CARS signal was isolated from the excitation laser wavelengths by an optical filter set composed of a 950 SP (short pass), a 785 SP, two 775 SP, and a 514 LP (long pass) filter (all Semrock, United States) and a 650/40 BP (bandpass) filter. Detection of the CARS signal is accomplished by a photomultiplier tube (PMT (H 9656-20, Hamamatsu Photonics, Japan). The resulting electronic signal was acquired by an analog-to-digital (A/D) converter (PCI-6110S, National Instruments, United States) and used for visualization by the MATLAB program ScanImage (version 3.8.1, Howard Hughes Janelia Farm Research Campus). For SRS imaging the Stokes beam was modulated by a resonant electro-optic modulator operating at 20 MHz. For the acquisition of the SRS signal the pump beam was isolated from all other wavelengths by the 950 SP and an 800/50 BP (Chroma) filters and was directed onto a customized photodiode. The electronic signal was filtered and demodulated in combination with the 20 MHz reference signal by a specifically adapted lock-in amplifier (APE, Berlin), which couples the demodulated SRS signal into the A/D converter card. For CARS imaging the 2845 cm^–1^ molecular resonance was probed to visualize the lipid distribution in the sample. The focal intensities were set for the Stokes beam (1032 nm) at 16.5 mW and for the pump beam (799.3 nm) at 33 mW, respectively. For second harmonic generation (SHG) imaging the laser source was switched to a home-built fiber-based femtosecond laser with 400 fs pulse length (55MHz repetition rate) (described in Kong et al., 2017) operating at 1054 nm using 20 mW focal intensity while the filter set was changed to a bandpass at 532/18 nm and the 950 SP as well as two times the 785 SP to isolate the SHG signal.

### Confocal Microscopy

In preparation for confocal microscopy, human liver tissue was fixed in 4% formaldehyde/PBS for 2 h, embedded in 6% low melting point agarose and 100 μm thick vibratome sections were prepared. The sections were subsequently permeabilized (0.5% Triton X-100/PBS) and blocked in Permblock solution (3% BSA, 0.5% Tween-20 in PBS). Antibodies used have been described under whole mount preparations. Sections were incubated overnight at 4°C in primary antibody dilution, washed three times with PBS-T (0.1% Tween-20/PBS) and finally incubated in secondary antibodies for 1 h at room temperature. The stained samples were mounted with Mowiol and imaged using a commercial laser scanning fluorescence microscope (Zeiss LSM880, 20x, NA = 0.8).

### Super-Resolution Structured Illumination Microscopy (SR-SIM)

Human LSEC were imaged using a commercial super-resolution structured illumination microscope (SR-SIM) (DeltaVision| OMXv4.0 BLAZE, GE Healthcare) equipped with a 60 × 1.42NA oil-immersion objective lens (Olympus). 3D SR-SIM image stacks of 1 μm thickness were acquired with a vertical distance between image planes of 125 nm and with 15 raw images per plane (five phases, three angles). Raw datasets were computationally reconstructed using the SoftWoRx software (GE Healthcare). For clarity of display, figure images were linearly adjusted for brightness and contrast using Fiji (^2^ version 2.0.2.) (Schindelin et al., 2012).

## Results and Discussion

Optical imaging methods offer a highly “natural” way of analyzing tissue samples because they extend and exploit the evolutionarily optimized strong human visual perception and have therefore been employed by scientists for centuries. The combination of particularly gentle preparation techniques (in comparison to the sample preparation required for electron-microscopy) with label–free imaging or genetically encoded reporters, such as fluorescent proteins, allow the application of optical methods under conditions that preserve the sample in its most natural state, where even mechanical and morphological sample properties such as elasticity, size and shape are mostly maintained. It should be mentioned that this statement is no longer correct if fixation, permblock or dehydration and refractive index matching are used as part of the sample preparation, in which case control experiments need to be considered in order to ensure that sample preparation does not interfere with the conclusions drawn from optical measurements. A particular strength of optical imaging modalities is, however, that they benefit from a wide range of contrast methods that have been developed, in particular during the last couple of decades. Fluorescence microscopy is an especially attractive method, because fluorescent staining of samples provides highly specific molecular contrast. This is achieved either through the use of organic fluorophores, which bind specific molecular structures within the sample (e.g., lipophilic dyes will stain lipids, intercalating dyes will stain chromatin or nucleic acids, etc.) or through incubation of the sample with highly specific binders such as antibodies or nanobodies to which fluorophores have been coupled. An approach intensely developed in the recent past is the expression of molecular tags with the capacity to bind fluorophores or to convert non-fluorescent dyes to a fluorescent form. In the following, we demonstrate how excised and fixed samples of the human liver can be visualized and analyzed by optical microscopy methods from the millimeter scale down to the nanometer scale. We provide examples for mesoscale, microscale and nanoscale fluorescence microscopy methods and their partial combination that allow for comprehensively imaging liver morphology. In addition, we demonstrate how these methods can be further enhanced by the introduction of highly specific label-free chemical imaging techniques.

### Mesoscopic Imaging of the Human Liver by Fluorescence Microscopy

**FIGURE 2:**
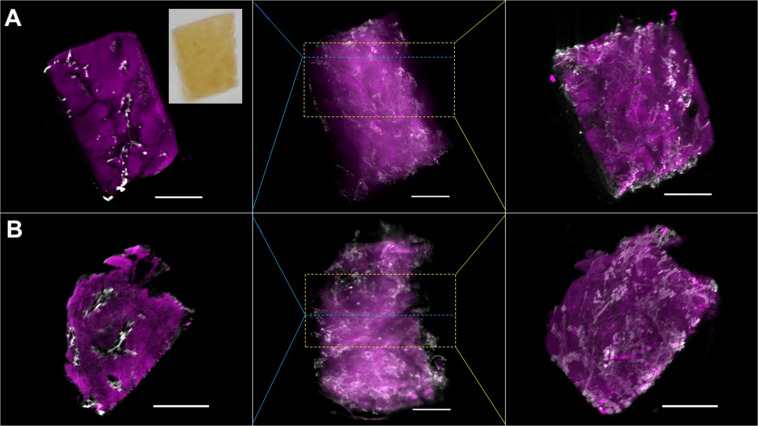
Optical projection tomographs (OPT) of the human liver. **(A)** and **(B)** are two different liver biopsies obtained from the same patient, each covering a volume of approximately 3 mm × 3 mm × 5 mm. The images in the center column panels are projections of the fluorescence from the entire sample. The images in the left panels show single cross sections that were taken at the regions indicated by the blue dashed lines in each row. The images in the right panels are projections of a stack of cross sections, the extent of which is indicated by the yellow dashed box. The specimen were stained with antibodies against a smooth muscle actin identifying smooth muscle cells (magenta, indicated by arrowheads in the individual panels) and cytokeratin 19 identifying bile ducts (white, indicated by asterisks in the panels) and subsequently cleared following the BABB protocol. The inset in the upper left panel shows a photograph of the optically cleared liver sample in BABB. Scale bars are 1 mm.

The scale on which a sample can be imaged and the spatial resolution, which is achieved by a particular method typically go hand-in-hand - at least if the acquisition times for the imaging process shall be kept reasonable. Thus, for imaging liver tissue on the millimeter scale, the spatial resolution is typically limited to the micron scale. A particularly attractive method for imaging entire millimeter sized liver samples that was developed within the last 2 decades is optical projection tomography (Sharpe et al., 2002). Optical projection tomography (OPT) is the optical analog to X-ray computed tomography. In OPT light is passed through a sample and an image is taken with a camera. The sample is then rotated in the light path at small inclinations and additional images are taken at every step until the sample was rotated by 180°, or better 360°. The images that were collected in this way could be either transmitted light images or fluorescence images and each pixel in an image represents the line integral of the chosen contrast projected through the sample. Three-dimensional images of the sample are then reconstructed using a filtered back-projection algorithm based on an inverse Radon transform of the data (Sharpe, 2004). This method does, however, require that light can be transmitted without significant absorption or scattering through the entire sample, which is rather difficult to achieve in the case of the liver. Thus, optical clearing methods are typically applied to generate optically transparent samples, with protocols based on organic solvents having the longest history. Organic solvents extract lipids from the sample and hydrate the tissue. In a subsequent step the water in the sample is replaced by a refractory index matching liquid, such that structures that lead to the absorption or scattering of light are largely removed or minimized (Orlich and Kiefer, 2018). The result of this is shown in Figure 2, where two liver biopsy specimens obtained from the same human donor were immunostained, subsequently optically cleared and then imaged by OPT. A representative photograph of one of these optically cleared liver samples is shown in the inset in the upper right corner of row A in Figure 2. Samples were whole mount immunostained for a smooth muscle actin (aSMA) and the intermediate filament cytokeratin 19. aSMA decorates smooth muscle cells, which are present as mural cells in the blood vessel walls, not notably in arteries and arterioles (shown as magenta in Figure 2). Cytokeratin 19, on the other hand, stains cholangiocytes, which results in highlighting the bile ducts (shown as white in Figure 2). As described in Materials and Methods, following delipidation and dehydration and refractive index matching in Murray’s clear (BABB), samples were mounted on a rotation stage and fully immersed in Murray’s clear within an imaging quality quartz cuvette for the imaging process. Fluorescence images were acquired sequentially for the different fluorophores.

Each of the samples in Figures 2A,B covers a volume of approximately 3 × 3 × 5 mm. A movie showing the 3D reconstruction of the OPT data is presented in Supplementary Video 1. Center panels in Figure 2 show projections of the fluorescence from the entire sample, left panels show optical cross sections at the level indicated by the blue dashed lines while the right panels depict projections of a stack of optical cross sections indicated by the yellow dashed rectangles. The aSMA staining revealed the distribution of liver arteries and veins, while the bile ducts were identified by cytokeratin. We noted a relatively high background of the aSMA staining, depicted in the magenta channel, which was augmented by intense aSMA signal from arteries and arterioles throughout the specimen. This effect becomes naturally most obstructive in the maximum intensity projections shown in the central panels. In order to obtain a more detailed, higher resolution volumetric view, the same samples were also imaged by light sheet fluorescence microscopy (LSFM). Over the last decade light sheet microscopy, originally perceived by Siedentopf and Zsigmondy (1902) more than a century ago, has undergone a vivid renaissance, which was originally sparked by the work of Stelzer and coworkers (Huisken et al., 2004). In contrast to the traditional fluorescence microscopy modalities such as epifluorescence, confocal and multiphoton microscopy, in LSFM the sample is not illuminated through the objective lens but orthogonally to the detection path by a thin sheet of light (Power and Huisken, 2017). This thin light sheet, generated by a scanned laser beam or more traditionally through shaping of a Gaussian beam using cylindrical lenses, is then exploited to scan the *z* axis of the sample in a stepwise fashion. Uncoupling of the illumination and detection light paths in this modality offers significant benefits. First, because fluorescence within the sample will only be excited in the volume illuminated by the light sheet, the thickness of the sheet defines the focal planar volume and hence z axis resolution. Therefore, further measures to suppress undesired out-of-focus fluorescence are not required. Second, because the sheet forming optics, which can be freely configured, defines the resolution in z, the lateral resolution of the detection optics can be matched to the sheet such that the resulting point spread function is close to isotropic. Third, the entire focal plane can be imaged simultaneously using an area detector (scientific camera), making image acquisition in light sheet microscopy significantly faster as e.g., scanning modalities such as confocal microscopy that rely on photomultipliers. Fourth, because only the actual volume being detected is illuminated, LSFM does not require illumination throughout the entire sample, which significantly reduces phototoxicity in live imaging and photobleaching of fixed, stained samples (Mertz, 2011).

While originally conceived for the analysis of colloidal dispersions in glass, an obvious limitation of LSFM is the requirement for nearly complete tissue transparency, which presently limits the technology to either transparent small model organisms such as zebrafish larvae or tissue samples that have undergone optical clearing. The roots of tissue clearing also date back longer than a century. A plethora of new and innovative approaches have been developed and refined over the last decade, described in a number of excellent recent reviews on the topic (Costantini et al., 2019; Matryba et al., 2019).

**FIGURE 3:**
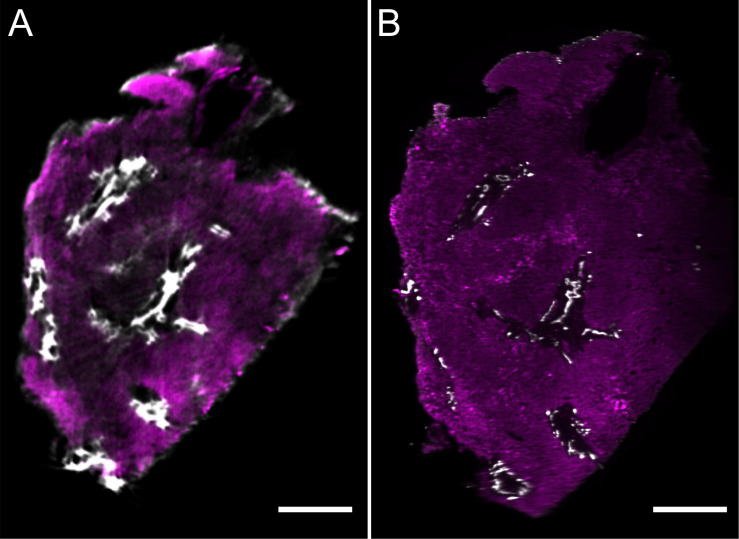
Comparison of liver volumes imaged by optical projection tomography **(A)** and light sheet microscopy **(B)**. Optical sections of the same wholemount immunostained liver biopsy (for antibodies and colors also see Figures 2, 4) were acquired with both optical projection tomography **(A)** and light sheet microscopy **(B)**. Image stacks were visualized using the volume rendering software package Voreen. Subsequently, rendered volumes were digitally oriented such that the sectional planes were approximately matching and virtually isolated, thin optical section corresponding to 9.6 μm **(A)** and 5 μm **(B)** are shown for direct comparison. Arrowheads identify corresponding structures. Scale bars represent 500 μm.

The results reported here were obtained with a basic single objective configuration, illuminated by a dual sided light sheet and we visualized image stacks using our proprietary volume-rendering framework Voreen (Meyer-Spradow et al., 2009; Dierkes et al., 2018). We analyzed the same immunostained liver biopsy specimen with LSFM that was later also imaged using OPT. A movie showing the 3D reconstruction of the LSFM data is shown in Supplementary Video 2. Figure 3 shows a direct comparison of two corresponding planes of this sample imaged by OPT (Figure 3A) and LSFM (Figure 3B). Both technologies assess mesoscopic tissue volumes, hence corresponding tissue planes were identified *in silico* from the digitally 3D rendered volume representations. As can be seen from this comparison, both techniques provided an excellent representation of the bile ducts but also identified blood vessels in the surrounding tissue, albeit the staining contrast over background was less pronounced for these structures. As expected, OPT provided a somewhat lower spatial resolution, however, the specimen only occupied a fraction of the maximum imaging volume that can be assessed with this instrument. By adjusting the magnification of the lenses used in the OPT, the resolution could be matched to that of LSFM, but the main purpose of the instrument in its current state is to provide volumetric imaging of tissues that are typically too large to be imaged by LSFM, where the thickness to which a sample can be imaged depends mostly on the working distance of the objective lenses.

**FIGURE 4:**
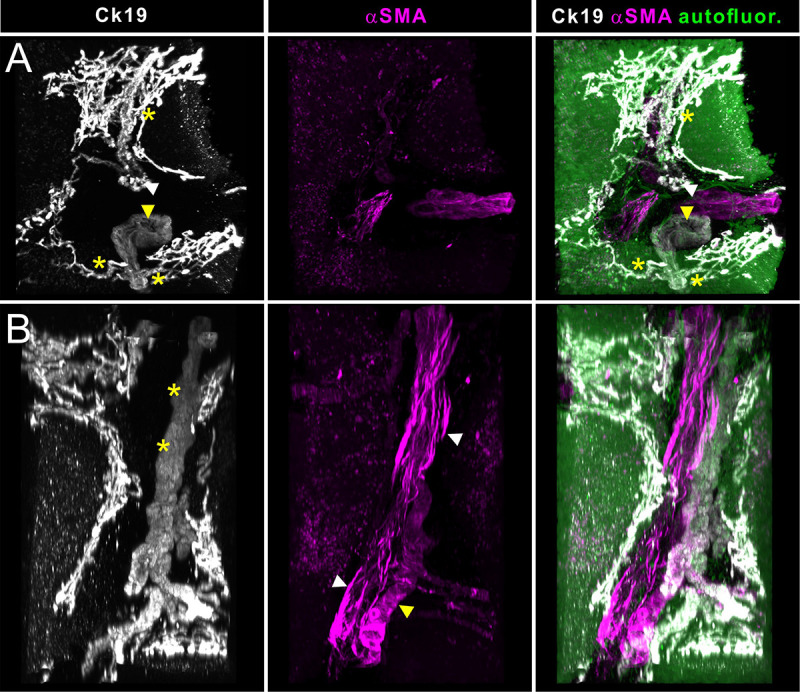
Light sheet fluorescence microscopic analysis of human liver biopsies. Whole mount human liver biopsies stained for smooth muscle actin (magenta) and cytokeratin 19 (white) – identical specimen as depicted in Figures 2, 3. Tissue autofluoresence (green) provided anatomical landmarks. Shown are maximum intensity projections of a tissue volume of approx. 1500 μm × 1300 μm × 800 μm. **(A)** Cross sectional aspect of a hepatic portal field. Besides the supplying blood vessels, shown in magenta in the central panel (likely parts of the portal venous connection), a smaller (white arrowhead) and a larger bile duct (yellow arrowhead) and the reticular network formed by their upstream smallest bile ductuli (Ducts of Hering). Communicating ducts between this reticular networks and the portal ducts are marked by asterisks. **(B)** Volume rendering showing a longitudinal aspect of the vasculature and bile ducts running in a portal field. Note the presence of two supplying blood vessels, which show distinct differences in the orientation of the smooth muscle cells in the vessel wall (central panel, magenta). Longitudinally running smooth muscle cells identify branches of the venous vasculature (white arrowheads), while a circumferential orientation of the smooth muscle cells is indicative of arterial vessels (yellow arrowheads). Cytokeratin staining is gradually downregulated in the more differentiated cholangiocytes of the larger caliber bile ducts (asterisk).

A particular strength of volumetric imaging of whole mount stained samples by OPT and LSFM is the minimized risk that rare events go unnoticed, which is a permanent danger in section-based approaches. Quantitative analysis based on this type of volume imaging usually gains enormously in statistical power over section-based analysis because the frequency of analyzed events is significantly increased. This is demonstrated in Supplementary Video 1 for OPT data and Supplementary Video 2 for LSFM data. In addition, the possibility to digitally reorient the sample freely on a personal computer is invaluable during structural and anatomical analysis. This is demonstrated in Figure 4, where portal regions can be effortlessly inspected in a cross sectional and a longitudinal view. In the latter, we were able to identify the affiliation of the blood vasculature with the arterial or portal venous tree based on the smooth muscle cell orientation (see Figure 4B, white arrowheads). The 3-dimensional structure of the smallest bile ducts originating at the hepatocyte canaliculi (Ducts of Hering) unexpectedly revealed a reticular network, often originating with bulbous small ductal stubs. Patches of this network then communicate via a single or few ductuli with the portal ducts.

### Microscopic Imaging of the Human Liver by Fluorescence and Label-Free Confocal Optical Microscopy

**FIGURE 5:**
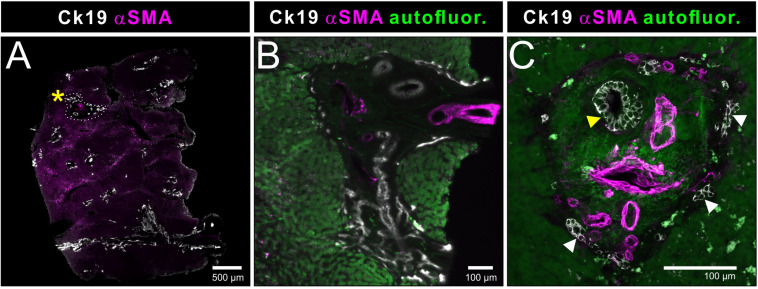
Light sheet and confocal imaging of two representative human liver portal fields. **(A)** Light sheet microscopic overview of an immunostained human liver biopsy, in which specific structures of interest, here a prototypic portal field, are easily identified (encircled area next to asterisk). **(B)** Magnification of the cross section of one portal field from the rendered volume shown in **(A)**. **(C)** Confocal image of a portal field from a 100 μm section identically stained to the specimen depicted in Figures 2, 4. Note the size difference of the cholangiocytes between the smallest bile ducts (white arrowheads) and the larger duct but also within the section of the larger bile duct (yellow arrowhead).

The large overview images provided by previously described mesoscopic imaging techniques make the identification of particular structures of interest straightforward. This is illustrated in the LSFM volume in Figure 5A where a portal region can readily be identified. Closer inspection at higher resolution then reveals the hepatocyte trabeculae of the adjacent lobule blood vessels (shown in magenta) and bile ducts (shown in white, see Figure 5B). The spatial resolution of LSFM is sufficient to identify the lumen even in the small Canal of Hering at the edge of the portal field and to discern individual cholangiocytes in the bile ducts. Confocal microscopy (Pawley, 2006), which for one-photon excited fluorescence is limited to sections of at most 100 – 200 μm thickness and small fields of view in the absence of image tiling, clearly surpasses the resolution achieved by mesoscale LSFM and allows us to reveal subcellular structures of the cholangiocytes and the fibers of the portal field extracellular matrix (ECM, see Figure 5C). This level of resolution is well complemented by non-linear optical imaging methods, such as multi-photon excited fluorescence, coherent Raman scattering, or higher harmonics generation within the sample, which, because these techniques utilize short laser pulses with wavelengths in the deep red to near infrared spectral region, can penetrate even deeper into the sample. This extended penetration depth is attributed to a lower absorption and less scattering of light within this spectral region in tissue.

**FIGURE 6:**
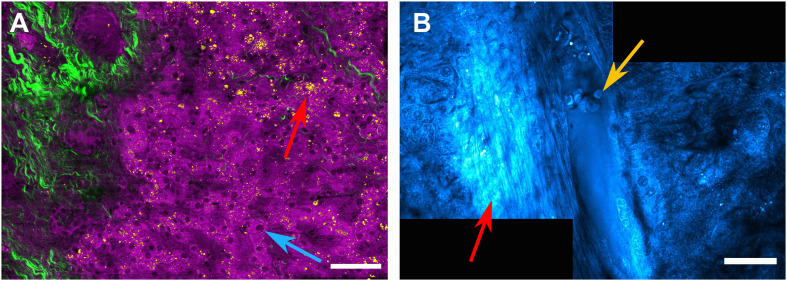
Non-linear optical confocal microscopy of human liver biopsies. **(A)** CARS image probing the 2845cm^– 1^ lipid resonance. By signal thresholding, weak background signals depicting single hepatocytes and their nuclei (blue arrow) are shown in magenta and can be separated from lipid droplets, which provide signal above the threshold value (red arrow), shown in yellow. The green color depicts parts of the sample producing a SHG signal, which was acquired subsequently utilizing a femtosecond fiber-laser source. SHG indicates fibrous structures within the liver tissue. Scale bar is 50 μm - note that several images are stitched together to obtain a larger field of view. **(B)** CARS image (at 2845cm^– 1^) of a portal vein with erythrocytes attached to the lumen (yellow arrow). In the vicinity of the vein, fibrotic alterations of the tissue can be seen (red arrow). Scale bar is 30 μm.

Raman scattering, i.e., the inelastic scattering of photons by molecular bonds is a particularly interesting alternative to fluorescence excitation as it provides intrinsic, label-free chemical contrast of biological samples (Huser and Chan, 2015). Spontaneous Raman scattering by molecular bonds is a rather weak process due to its inherently low scattering cross section, but it can be significantly enhanced by coherent Raman scattering. Here, highly focused, short laser pulses specifically probe molecular vibrations of interest by a pump-probe type process. A pump photon initially prepares the samples for Raman scattering. Molecular bonds in the sample then interact with a simultaneously arriving probe photon in a process called four-wave mixing if the wavelength difference between the probe photon and the pump photon corresponds to the molecular vibration of interest. This interaction results in the emission of either a blue-shifted anti-Stokes photon in a process called coherent anti-Stokes Raman scattering (CARS) or in the emission of an additional photon with the same wavelength as the pump or the probe photon (stimulated Raman scattering, SRS). Over the last decade, this method has gained considerable interest in the biomedical sciences and is now frequently used to image e.g., lipid deposits in tissues *in vitro* and *in vivo*, and even to generate virtual H&E staining contrast for *in vivo* pathology (Cheng and Xie, 2015). Here, we demonstrate how this process can be used for the analysis of human liver tissue on the scale of hundreds of microns. By combining this chemically specific imaging methodology with second harmonic generation (SHG), initiated by femtosecond laser pulses, contrast for fibrous structures can also be gained, further enhancing the range of label-free contrast methods. Due to the non-linear nature of the signal generation, CARS/SRS and SHG are confined to the focal region of the laser beams, resulting in a typical spatial resolution of < 400 nm and excellent optical sectioning capabilities in the axial direction in the range of > 600 nm. Thus, this intrinsic confinement of the signal generation permits three-dimensional imaging with little to no background signal. Figure 6A shows a large area scan of a liver tissue section with 40 μm thickness, where CARS was used as contrast mechanism. Here, the 2845 cm^–1^ CH_2_ stretching mode, which is predominantly associated with aliphatic lipid vibrations, is probed. By utilizing simple signal thresholding, the signal contribution is divided into signals below the threshold value (shown in magenta), which are typically due to a CARS-inherent non-resonant background and possibly lower lipid content in membranes and proteins. This signal enables us to visualize hepatocytes (Figure 6A), revealing also the position of their nuclei (blue arrow), and the sinusoids in between. The higher signal contribution above the threshold value is shown in yellow and allows us to identify lipid droplets (red arrow, Figure 6A) within the tissue. Subsequently, SHG imaging is performed as an additional contrast mechanism applied to the same sample area and overlaid in green, highlighting collagen structures due to the frequency doubling of a femtosecond fiber laser source. This contrast reveals fibrotic tissue sections within the liver tissue. In order to obtain an even larger field of view, several CARS images were acquired by automatic sample movement using a motorized stage and subsequent stitching of the individual images to obtain the large area view shown in Figure 6B. Here, the CARS image (still probing the CH_2_ lipid resonance) reveals a portal vein with erythrocytes attached to the vessel wall (yellow arrow). In the vicinity of the vein, fibrotic tissue can, again, be seen (red arrow, Figure 6B).

**FIGURE 7:**
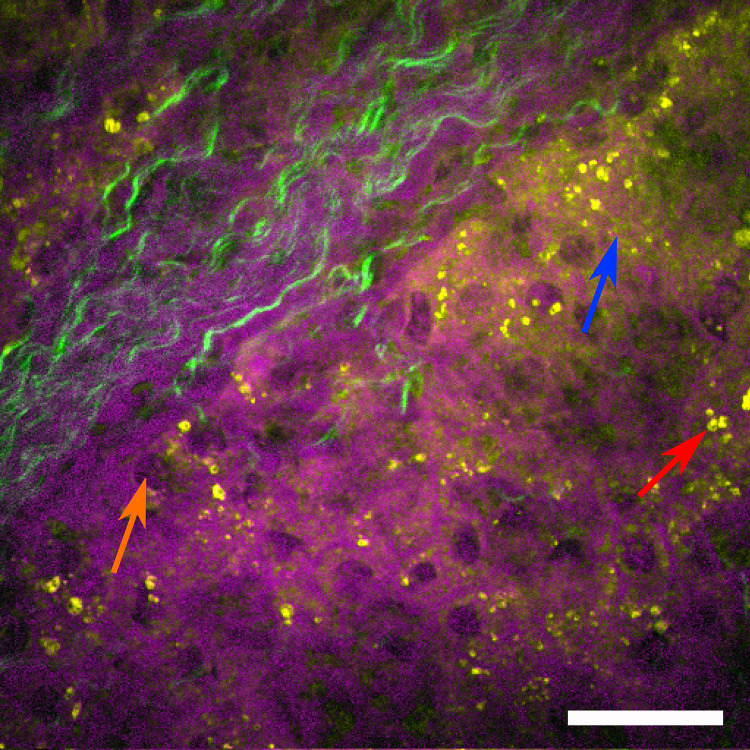
Hyperspectral SRS image of human liver biopsy. Hyperspectral SRS image probing the molecular resonances from 2790 to 3020 cm^– 1^ (utilizing spectral fitting for every pixel, where amplitude values of the peaks at 2845 cm^– 1^ (indicating lipids) are shown in yellow and at 2920 cm^– 1^ (indicating proteins) are shown in magenta. Single hepatocytes as indicated by their nuclei (orange arrow), sinusoids as well as lipid droplets (red arrow) can be identified. The green color channel shows the SHG signal indicating fibrotic tissue, which extends from the portal tract into the liver parenchyma. The parenchyma shows microvesicular steatosis (highlighted by a blue arrow). Scale bar is 30 μm.

To further improve the separation of nuclei and other cellular components, a more sophisticated method, called hyperspectral imaging, can be applied to sample areas of interest previously identified by the faster single resonance CARS microscopy (Cheng and Xie, 2015; Pilger et al., 2018). In hyperspectral imaging, not only a single molecular resonance is probed, but resonances within an entire wavelength range are acquired by automated wavelength tuning of the pump beam source. Here, the range of 2790 to 3020 cm^–1^ was scanned with a step size of 15 cm^–1^, covering two CH_2_ stretching resonances at 2845 cm^–1^ (symmetric stretching, depicted in yellow) as well as the 2920 cm^–1^ resonance (anti-symmetric stretching mode, depicted in magenta), which highlights lipid and protein contributions, respectively. In addition, the contrast mechanism was changed from CARS to SRS, which is technically a more challenging approach, but offers the advantage that it does not contain non-resonant background contributions, which are intrinsic to the CARS signal generation process. Once a stack of images has been acquired, where for each image the pump beam was spectrally shifted by 15 cm^–1^, an SRS spectrum can be generated for each pixel of the image. By fitting preselected Raman spectra to the data set, a false-color hyperspectral SRS image (Figure 7) can be generated, where specific colors are assigned to individual Raman resonances. Single hepatocytes with their nuclei (orange arrow) as well as an extended amount of lipid droplets (red arrow) can be identified in the liver tissue. Again, the SHG signal was subsequently acquired to highlight the fibrotic regions within the sample (green), which extends from the portal tract into the liver parenchyma, while the parenchyma exhibits microvesicular steatosis as indicated by the accumulation of a large number of lipid droplets (yellow).

### Super-Resolution Structured Illumination Microscopy of Human Liver Sinusoidal Endothelial Cells

**FIGURE 8:**
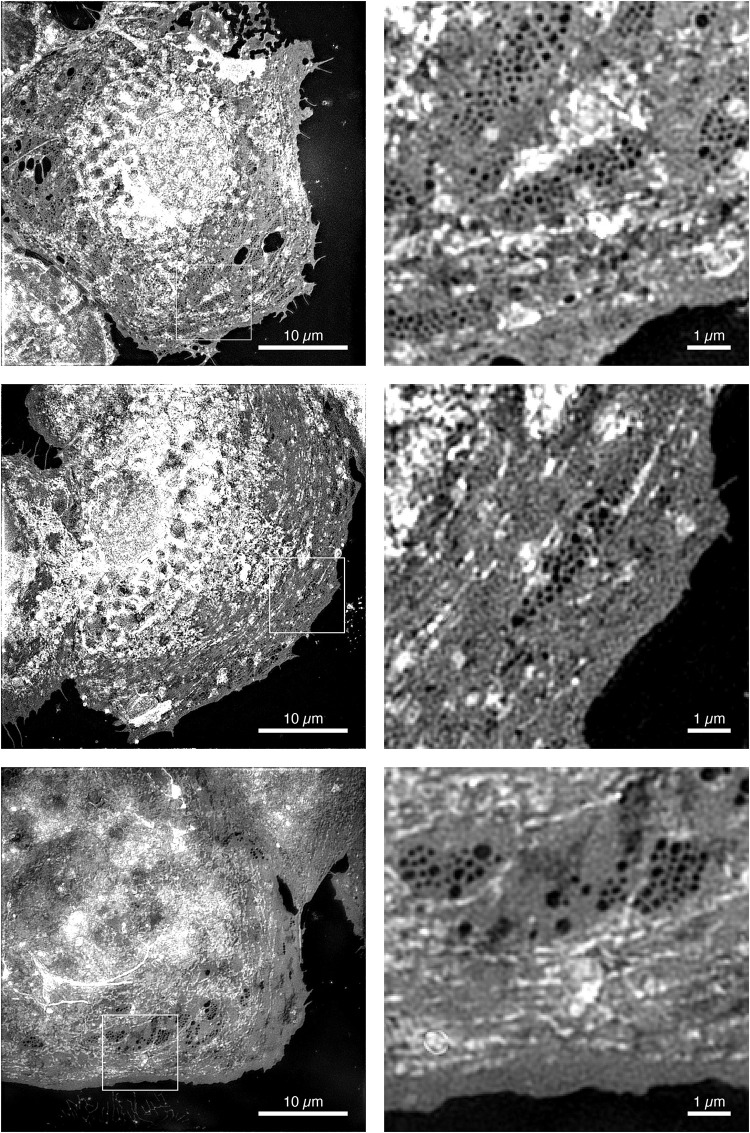
Super-resolution structured illumination micrographs of human LSECs. Super-resolution structured illumination microscopy images of 3 different human liver sinusoidal endothelial cells. The cells were stained with the membrane dye CellMask Orange, which allows the visualization of fenestrae as dark holes. The first column shows images with a full field-of-view of 40 μm for each cell. The second column shows magnified views of the white outlined boxes in the images to the left typically displaying one or more sieve plates.

The first description and electron microscopic observation of LSEC fenestrae was given by Wisse (1970). Today, half a century later, advanced optical microscopy techniques have evolved which allow us to resolve these nanoscale features in fresh, live and/or fixed cultures of cells (Schermelleh et al., 2019). Super-resolution structured illumination microscopy (SR-SIM) is a super-resolution microscopy (SRM) technique that is particularly attractive for imaging LSEC (Gustafsson et al., 2008; Schermelleh et al., 2008). In the linear implementation of SR-SIM, rather than illumination the sample with an even, homogeneous illumination, an interference pattern is created within the sample. If the interference pattern has a periodicity close to the smallest spatial feature that can be resolved by the microscope objective lens, then SR-SIM achieves approximately twice the spatial resolution obtained with high-resolution conventional fluorescence microscopy, i.e., approximately 100 nm laterally (Heintzmann and Huser, 2017). Alternatively, by creating a sinusoidal interference pattern with optics using high index of refraction materials, a spatial resolution below 90 nm was demonstrated (Li et al., 2015). In a non-linear implementation, where rather than a slowly varying sinusoidal illumination, a pattern with steep edges is created in the sample and a spatial resolution of less than 50 nm has been achieved (Gustafsson, 2005; Rego et al., 2012; Li et al., 2015). Currently, however, non-linear SR-SIM requires either saturating the fluorescence excitation or the use of photoswitchable fluorophores (Gustafsson, 2005; Rego et al., 2012). Both of these restrictions currently prohibit their use with LSECs: saturating fluorescence requires high laser power, which is detrimental to cell health and the fluorescence photobleaches rapidly. And the genetic modifications required to incorporate photoswitchable fluorescent proteins could, at best, only be done with animal models and are not possible with human LSEC. A last issue is that LSEC fenestrae cannot be labeled directly and, instead the plasma membrane needs to be stained. Linear SR-SIM, on the other hand, does not require specific properties of fluorescent probes and works with most fluorophores, it can easily be extended to multiple colors (Schermelleh et al., 2008) and it is fast - enabling even the imaging of living cells at video rate (Markwirth et al., 2019). The ∼ 100 nm lateral spatial resolution is sufficient to visualize fenestrations in LSEC as was originally shown in fixed rat LSECs (Cogger et al., 2010; Svistounov et al., 2012). Here, we demonstrate this ability by imaging fenestrations in human LSEC (hLSEC). In order to visualize fenestrae, the plasma membrane of fixed hLSECs was stained with an orange-fluorescent membrane stain. The result can be seen in the series of images shown in Figure 8. Here, 3 hLSEC out of a series of > 50 cells that were imaged in a single session are shown, which clearly exhibited groups of fenestrae organized in so-called sieve plates. Enlarged versions of such sieve plates are shown next to the full cell SR-SIM images and correspond to the regions of interest outlined by dashed white squares. As can be seen from these images, sieve plates occur primarily in the extended parts of the plasma membrane far from the nucleus, where the distance between the basal and apical membrane is typically the thinnest. Also, in stark contrast to rat LSECs, where sieve plates can occupy up to 60% of the entire cell’s surface (Mönkemöller et al., 2015), a significantly smaller fraction of the membrane of hLSEC is covered by sieve plates. We attribute this to defenestration due to the old age of the human patient from which these samples were obtained, as well as potentially underlying health conditions affecting the health of these rather sensitive cells (Couteur et al., 2008).

**FIGURE 9:**
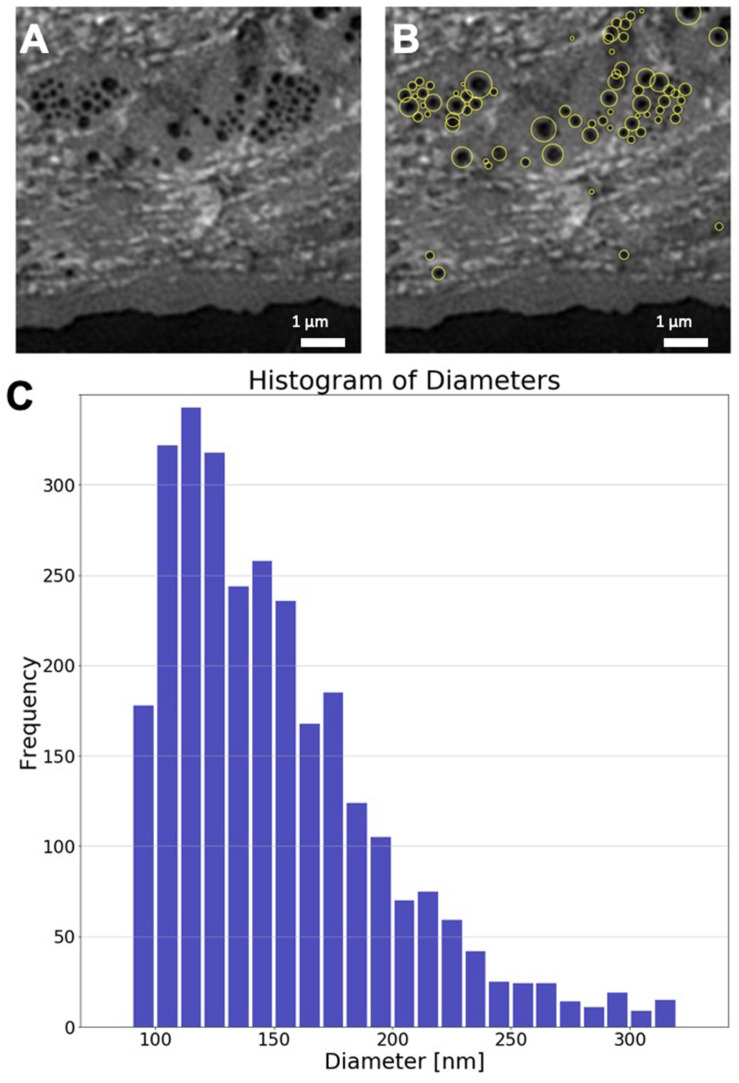
Analysis of hLSEC fenestration diameters. **(A)** Region of interest of an SR-SIM image depicting several sieve plates in the plasma membrane of hLSEC. **(B)** The same image as shown in **(A)**, where fenestrae that were automatically identified and sized are highlighted by yellow circles, where the circle diameter corresponds to double the diameter identified for each fenestra. **(C)** Size distribution histogram of 4471 fenestrae identified in 21 regions of interest taken from 5 hLSECs.

In order to obtain a quantitative measure of the size distribution of fenestrae in hLSEC, we identified and measured fenestrae in 5 different hLSEC using an automated image processing macro written in Python. Previous distributions of fenestration sizes were obtained by hand and the automation of this process can be difficult because of the significant variations in local brightness due to uneven staining of the cells, as is apparent from the images shown in Figure 8. The Python macro utilizes an adaptive thresholding process to identify fenestrae. Specifically, images are first opened, expanded to double the original pixel count, and then dilated with a filter corresponding to the doubled pixel number. This process evens out the brightness distribution. The image is then inverted and the adaptive thresholding process applied. Here, the user can choose the threshold value, which needs to be adjusted from image to image. Subsequently, local maxima are found, and adjacent, potentially overlapping fenestrae are separated by watershedding. Finally, the fenestrae are segmented and measured. Fenestrae with a diameter < 95 nm were excluded, because of the spatial resolution limit of SR-SIM, and fenestrae with a diameter > 320 nm were also excluded, because these are considered to be holes in the membrane rather than fenestrae. The outcome of this process is demonstrated on the example region of interest shown in Figures 9A,B. Here, the same region of interest as shown in the lowest row of Figure 8 was selected and the fenestration finding macro was applied to the region of interest. Figure 9B shows fenestrae that were identified by this macro highlighted by yellow circles. The diameter of these circles highlighting fenestrae corresponds to double the measured diameter, which enables the easier identification of the underlying fenestrae by the human eye. The diameters are also written into a text file. We have applied this process to a total of 21 regions of interest selected from the 5 human LSECs where sieve plates were most clearly visible. This resulted in 4471 fenestrae being identified. Their size distribution is shown in the histogram in Figure 9C. As can be seen from this histogram, in the human LSEC the distribution of fenestrae diameters is falling off exponentially in the range between 90 – 320 nm and it peaks in the range between 110 – 120 nm. This presents the first measurement of fenestration diameters in hLSEC by SRM under aqueous conditions.

## Conclusion

In conclusion, we have demonstrated the optical imaging of liver morphology and liver ultrastructure across 7 orders of magnitude. Mesoscopic imaging techniques, such as optical projection tomography and light sheet fluorescence microscopy were used to produce three-dimensional maps of liver tissue. These methods allowed us to image the bile ducts, as well as blood vessels in optically cleared liver tissue with dimensions of a few millimeters by highly specific fluorescence contrast. The spatial resolution of LSFM was sufficient to identify the affiliation of the blood vasculature with the arterial or portal venous tree based on smooth muscle cell orientation. Remarkably, the smallest bile ducts originating at the hepatocyte canaliculi revealed a reticular network which extends a single or few ductuli toward the portal ducts. LSFM even enabled us to image the lumen in the small bile ductules at the edge of the portal field and to identify individual cholangiocytes in the bile ducts. Confocal fluorescence microscopy then seamlessly extended the spatial resolution to the subcellular scale and allowed us to image the inner structure of cholangiocytes and fibers of the portal field extracellular matrix. Label-free confocal microscopy, in particular a combination of coherent Raman scattering (CRS) together with second harmonic generation, allowed us to image a portal vein with erythrocytes attached to the vessel wall as well as nearby fibrotic tissue without fluorescent staining. Hyperspectral CRS imaging was then used to identify single hepatocytes and microvesicular hepatosteatosis based on the accumulation of lipid droplets, as well as fibrotic liver tissue which extended from the portal tract into the liver parenchyma. Lastly, the submicroscopic structure of human liver sinusoidal endothelial cells was imaged by super-resolution structured illumination microscopy. This allowed us to identify hLSEC with fenestrations and to determine the size distribution of fenestrae in hLSEC, which were measured to exhibit the largest fraction of diameters in the range between 110 – 120 nm. Even imaging of the fenestration dynamics of living hLSECs should be possible with linear SR-SIM, because of the availability of live cell plasma membrane stains. Extensions of the methods presented here, such as the combination of light sheet fluorescence microscopy with super-resolution optical microscopy will, in the near future, enable us to combine many of the different methods discussed here within a single instrument. This combination is expected to allow us to image the ultrastructure of the liver in extended tissue and it will further improve the quantitative imaging of veins, bile ducts, and sinusoids and our detailed understanding of their connections.

## Data Availability Statement

The datasets generated and analyzed for this study can be found on Zenodo.org using the DOI: 10.5281/zenodo.4300689.

## Ethics Statement

The studies involving human participants were reviewed and approved by Ethics Committee Münster, Germany, 2017-522-f-S. The patients/participants provided their written informed consent to participate in this study.

## Author Contributions

CK, SB, CP, ML, CØ, VM, and WH acquired data. All authors helped analyze the data. All authors were involved in writing the manuscript. FK, TH, and JS perceived the project and supervised the work.

## Conflict of Interest

The authors declare that the research was conducted in the absence of any commercial or financial relationships that could be construed as a potential conflict of interest.

## Footnotes

1 https://lag-opt.github.io/

2 https://fiji.sc
